# Premalignant lesions, basal cell carcinoma and melanoma in patients with cutaneous squamous cell carcinoma

**DOI:** 10.1007/s00403-020-02114-w

**Published:** 2020-08-09

**Authors:** Niina Korhonen, Leea Ylitalo, Tiina Luukkaala, Julius Itkonen, Henni Häihälä, Juha Jernman, Erna Snellman, Johanna Palve

**Affiliations:** 1 Department of Dermatology and Allergology, Tampere University Hospital and Faculty of Medicine and Health Technology, Tampere University, Tampere, Finland; 2 Department of Dermatology, Skin Cancer Unit, Helsinki University Central Hospital, Helsinki, Finland; 3 Research, Development and Innovation Center, Tampere University Hospital and Health Sciences, Faculty of Social Sciences, Tampere University, Tampere, Finland; 4 Department of Pathology, Tampere University and Fimlab Laboratories, Tampere, Finland; 5 Department of Plastic Surgery, Tampere University Hospital and Faculty of Medicine and Health Technology, Tampere University, Tampere, Finland

**Keywords:** Cutaneous squamous cell carcinoma, Basal cell carcinoma, Cutaneous melanoma, Actinic keratosis, Bowen’s disease

## Abstract

The incidence of keratinocyte carcinomas is increasing worldwide and currently there is no standardised strategy for the follow-up of patients with multiple tumours. The objective of this study was to assess the prevalence of premalignant lesions, i.e., actinic keratosis and Bowen’s disease, as well as basal cell carcinoma (BCC) and cutaneous melanoma (CM) among patients with cutaneous squamous cell carcinoma (cSCC). Pathology database search was performed to identify all cSCC patients diagnosed in the Pirkanmaa region of Finland in 2006–2015. Details of the patients and tumours were obtained through medical record review. The cohort consisted of 774 patients with 1131 cSCC tumours. Overall 559 patients (72%) had premalignant lesions. A total of 316 patients (41%) had BCC and 52% of these (*n* = 164) had more than one BCC tumour. 50 patients (6%) had CM. Overall 180 cSCC patients (23%) had no premalignant changes, BCC or CM. The median age of these patients was 6 years less than that of the patients with premalignant lesions (*p* < 0.001) or BCC (*p* < 0.001). The invasion depth of the tumours was deeper in the patients with only cSCC (median 3 mm, interquartile range 2–6) than in those with premalignant lesions or BCC (median 2 mm, interquartile range 1–3), *p* < 0.001. CSCC patients have a high risk of developing multiple skin cancers and need long-term follow-up.

## Introduction

The incidence of keratinocyte carcinomas, i.e., cutaneous squamous cell carcinomas (cSCC) and basal cell carcinomas (BCC), is increasing worldwide [1]. Although cSCC and BCC partially share similar risk factors [2, 3], they have fundamentally distinct and heterogeneous disease characteristics. CSCCs can emerge de novo but commonly originate from precursor lesions such as actinic keratosis (AK) and cSCC in situ, also known as Bowen’s disease (BD) [4]. There is a low risk of malignant progression for any given AK or BD and the presence of these precursor lesions predicts an increased risk of developing keratinocyte carcinoma or cutaneous melanoma (CM) compared with a matched population [5–7].

It has been reported previously that patients with keratinocyte carcinoma have a significantly greater risk of subsequent skin cancer [3, 8–11]. Less is known, however, about cSCC patients in particular and the impact of multiple cSCC tumours on the subsequent cancer risk has remained unknown. The aim of this study was to assess the prevalence of premalignant lesions (AK and BD), BCC and CM among cSCC patients in a Finnish regional university hospital cohort which included patients with multiple cSCC tumours.

## Materials and methods

To identify all the cSCC patients in the Pirkanmaa region of Finland, a pathology database search was performed at Fimlab Laboratories for histopathological diagnoses of ‘cutaneous squamous cell carcinoma’ (cSCC) between 1 January 2006 and 31 December 2015. In 2006, the population of Pirkanmaa was 473,490 at the beginning of that period and 506,114 in the last year, 2015 [12]. We reviewed the clinical records of Tampere University Hospital of all identified cSCC patients. This retrospective study was approved by the institutional review board of Tampere University Hospital, Finland.

Data obtained from the cSCC patients included patient age at diagnosis of the primary tumour, gender and associated diseases (particularly immunosuppression). Features of the cSCC tumours themselves, including anatomic location, degree of differentiation and depth of invasion, were also noted. Details of the cohort and time trends observable in it have been reported previously [13].

Patients were indicated as having AK, BD, BCC or CM if a prior history or current treatment until the end of the study period of such a lesion was recorded in the clinical notes. The total numbers of BCC and CM cases were collected for each patient. Patients were indicated as positive for AK and/or BD based on the clinical records, regardless of whether the lesion had been histologically confirmed or not. AK and BD were referred to as premalignant lesions distinguishing these from the invasive form of cSCC.

Categorical data were described by number of patients or number of tumours with percentages, and differences between such variables were tested with Pearson’s chi-square test or Fisher’s exact test. Due to the skew distributions, continuous variables were described by medians with interquartile ranges and tested with the Mann–Whitney test. All the tests were two-sided and a *p* value < 0.05 was considered significant, except that *p* < 0.001 was required for significance when Bonferroni correction was used in the interpretation of the results due to the multi-testing of AK/BD, BCC and/or CM. Statistical analyses were performed using IBM SPSS Statistics for Windows (version 23.0, Armonk, NY, USA, IBM Corp.).

## Results

After 567 diagnostic duplicates, i.e., overlapping data on biopsy and excision of the same case of cSCC, were excluded, the cohort consisted of 774 patients with 1131 cSCC tumours. A total of 81 (11%) patients were immunosuppressed, due to organ transplantation (*n* = 37), rheumatoid arthritis (*n* = 23), chronic leukaemia (*n* = 10) or lymphoma (*n* = 11).

### AK and BD in cSCC patients

Out of the 559 patients (72% of the cohort) who had premalignant lesions (AK and/or BD), 204 had only AK, and 46 only BD (Table 1). The remaining 309 patients had both. The median age of the cSCC patients with premalignant lesions was 82 years (interquartile range 76–86 years), and the prevalence of such lesions increased with age (Table 1).

**Table 1:** Patient characteristics and prevalence of premalignant lesions (actinic keratosis and/or Bowen`s disease, i.e., AK/BD), basal cell carcinoma (BCC) and cutaneous melanoma (CM) (patients *N* = 774)

	Total *N*	AK/BD (*n* = 559)		BCC (*n* = 316)		CM (*n* = 50)	
		*n* (%)	*p*	*n* (%)	*p*	*n* (%)	*p*
Sex			0.225		0.003		0.027
Men	394	277 (70)		181 (46)		33 (8)	
Women	380	282 (74)		135 (36)		17 (5)	
Age, years			< 0.001		0.003		0.368
< 60	35	11 (31)		4 (11)		1 (3)	
60–69	84	46 (55)		33 (39)		3 (4)	
70–79	238	175 (74)		95 (40)		21 (9)	
80–89	323	255 (79)		138 (43)		19 (6)	
≥ 90	94	72 (77)		46 (49)		6 (6)	
Immunosuppression			0.359		0.018		0.399
No	693	497 (72)		273 (39)		43 (6)	
Yes	81	62 (77)		43 (53)		7 (9)	
Number of SCC tumours			< 0.001		< 0.001		0.030
1	588	400 (68)		217 (37)		32 (5)	
2	116	95 (82)		51 (44)		8 (7)	
3	42	38 (91)		26 (62)		7 (17)	
≥ 4	28	26 (93)		22 (79)		3 (11)	

Differences between groups were tested using Pearson’s chi-square test or Fisher’s exact test

*N* total number of patients, *n* number of AK/BD, BCC or CM

The number of cSCC tumours recorded per patient during the study period ranged between 1 and 26, and patients with multiple cSCC tumours more commonly had premalignant lesions than those with a single tumour (Table 1).

If the tumour was located on the face (excluding lips, eyelids and ears) or upper extremity the patient was most likely to have premalignant lesions (Table 2). A total of 358 tumours had an invasion depth of 2 mm or less and in 85% of these cases the patient had premalignant lesions (Table 2). If the tumour was thicker than 4 mm, however, 55% of the patients had premalignant lesions. There was a tendency for patients with poorly tumour differentiation to have less premalignant lesions (Table 2).

**Table 2:** Characteristics of cSCC tumours and prevalence of premalignant lesions (actinic keratosis and/or Bowen`s disease, i.e., AK/BD), basal cell carcinoma (BCC) and cutaneous melanoma (CM) in the patient (tumours *N* = 1131)

	Total (*N* = 1131)	AK/BD (*n* = 880)		BCC (*n* = 554)		CM (*n* = 80)	
	*N*	*n* (%)	*p*	*n* (%)	*p*	*n *(%)	*p*
Tumour location							
Lip	52	37 (71)	0.237	15 (29)	0.003	2 (4)	0.576
Eyelid	11	6 (55)	0.074	4 (36)	0.400	2 (18)	0.180
Ear	106	77 (73)	0.179	57 (54)	0.300	9 (9)	0.550
Face	564	466 (83)	< 0.001	272 (48)	0.612	40 (7)	0.980
Scalp and neck	91	79 (87)	0.031	46 (51)	0.755	4 (4)	0.299
Trunk	61	51 (84)	0.262	40 (66)	0.008	4 (7)	1.000
Upper extremity	136	120 (88)	0.002	81 (60)	0.009	16 (12)	0.023
Lower extremity	68	39 (57)	< 0.001	34 (50)	0.863	3 (4)	0.622
Anogenital area	31	4 (13)	< 0.001	5 (16)	< 0.001	0 (0)	0.162
Oral cavity	11	1 (10)	< 0.001	0 (0)	0.001	0 (0)	1.000
Tumour differentiation			0.029/0.049		0.121/0.074		0.633/0.469
Well	521	402 (77)		272 (52)		34 (7)	
Moderate	450	357 (79)		205 (46)		31 (7)	
Poorly	89	60 (67)		39 (44)		9 (10)	
Unknown	71	61 (86)		38 (54)		6 (9)	
Invasion depth (in mm)			< 0.001/ < 0.001		0.071/0.040		0.751/0.595
< 1	45	42 (92)		25 (56)		4 (9)	
1.0–2.0	313	264 (84)		161 (51)		26 (8)	
2.1.0–4.0	203	149 (73)		98 (48)		11 (5)	
> 4.0	112	61 (55)		41 (37)		7 (6)	
Unknown	458	364 (80)		229 (50)		32 (7)	

Differences between groups were tested using Pearson’s chi-square test or Fisher’s exact test. P-values for tumour differentiation and invasion depth with/without unknown group

*N* total number of tumours, *n* number of AK/BD, BCC or CM

### BCC and CM in cSCC patients

Overall, 316 patients (41% of the cohort) had BCC (Table 1), these having a median age of 82 years (interquartile range 76–87 years). The number of BCC tumours per patient ranged from 1 to 19, with over half of the patients with BCC (*n* = 164, 52%) having more than 1 BCC tumour. Patients with multiple cSCC tumours during the study period were more likely to have BCC (Table 1).

A total of 50 patients (6% of the cohort) had CM (Table 1). The presence of CM was not associated with any specific location of cSCC tumour (Table 2), except that when the tumour was located on upper extremity the patient was slightly more likely to have CM than with some other location. The most common subtype of CM was superficial spreading melanoma (41%) while 18% were lentigo malignas, 10% in situ melanomas, 10% nodular melanomas, 2% lentigo maligna melanomas and 20% were not specified. The mean Breslow thickness was 1.3 mm.

### Combination of skin cancers

**Fig. 1:**
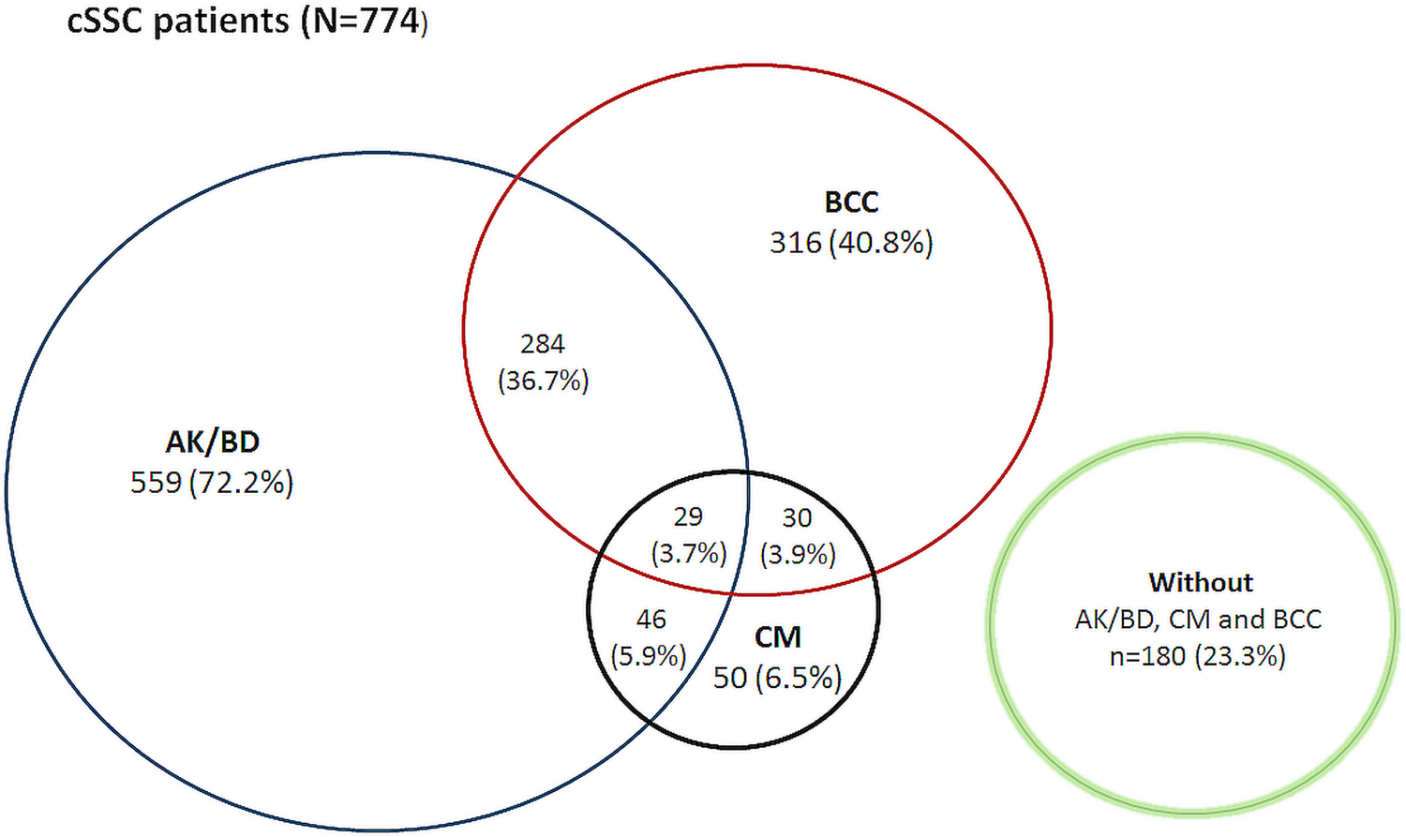
Combinations of tumour types among the cSCC patients. *AK/BD* actinic keratosis and/or Bowen’s disease, *BCC* basal cell carcinoma, *CM* cutaneous melanoma

A total of 284 patients (37%) had both premalignant changes and BCC (Fig. 1). CSCC patients with CM generally had premalignant changes (46/50 patients, 92%) and also commonly had BCC (30/50 patients, 60%). A total of 29 patients (4% of the cohort) had lesions of all types (premalignant lesions, BCC and CM) (Fig. 1).

Of all the 774 cSCC patients, 180 patients (23%) had no premalignant changes, BCC or CM (Fig. 1). The median age of these patients was 76 (interquartile range 66–84), i.e., they were 6 years younger than the patients with premalignant lesions (*p* < 0.001) and those with BCC (*p* < 0.001). Compared to with the patients with premalignant lesions, BCC or CM, these patients had less cSCC tumours per patient during the study period (*p* < 0.001), as 158 of them (88%) had only 1 tumour. The invasion depth of the tumours was deeper in the patients with only cSCC (median 3 mm, interquartile range 2–6, measured from 132 tumours out of 208) than in those with premalignant lesions or BCC (median 2 mm, interquartile range 1–3, measured from 541 out of 923 tumours), *p* < 0.001.

## Discussion

A total of 72% of the cSCC patients in this cohort had premalignant lesions and 44% had a different skin cancer (BCC and/or CM) in addition to cSCC. Since the patients with premalignant lesions commonly had multiple cSCCs and multiple BCCs, it seems that the diverse spectrum of keratinocyte carcinoma and its precursors accumulates in the same patients. The cSCC patients less often had CM, but in those cases they generally also had premalignant lesions and commonly BCC as well. It has been reported previously that a history of keratinocyte carcinoma is among the strongest risk factors for subsequent BCC, SCC and to a lesser extent CM [2, 3, 14, 15]. This suggests a partially shared aetiology and set of risk factors between different types of cutaneous malignancies which include exposure to UV radiation, genetic susceptibility and immunosuppression [3, 14, 16]. Furthermore, it has been reported that the risk of a subsequent new keratinocyte carcinoma with time is substantially lower after a first tumour diagnosis than after a non-first diagnosis [17]. The EDF–EADO–EORTC consensus group proposes follow-up clinical examinations for cSCC patients based on the risk profile of cSCC tumours [4]. For patients with multiple cutaneous malignancies and precursor lesions no consensus on the follow-up schedule exists.

The cSCC patients with premalignant lesions and BCCs were older than the others. It is known that the prevalence of AK increases with age, but it is likely that the prevalence has been underestimated [5]. Almost 38% of the participants in a Dutch population-based study (mean age 72 years) had one or more AK [18] and the prevalence of AK among people aged 70 or over in an Austrian study was over 50% [19]. It has been suggested that annual rates of progression from AK to cSCC range from 0% to 0.075% per lesion, with a risk of up to 0.53% per lesion in patients with a prior history of keratinocyte carcinoma [20]. Regardless of the grade of the lesions, it is not possible to predict which AKs will progress to invasive carcinomas [21]. Since over half of our patients with premalignant lesions had both AK and BD, this could mean that many patients had field cancerization, which could explain why multiple cSCC and BCC tumours emerged in these patients. There has been some debate about whether all AKs should be treated to prevent progression to cSCC [22] but at least individuals with numerous lesions are most likely to benefit from treatment and more frequent follow-up [5, 18] and this represents a growing future health-care challenge because of ageing populations.

The finding that the cSCC tumour was commonly located in an area exposed to the sun (face, scalp and neck or upper extremity) if the patient had premalignant lesions is line with the fact that cumulative lifetime exposure to UV radiation is the main risk factor for the development of cSCC [4, 23]. However, cSCC location in an area exposed to the sun was not closely associated with the prevalence of BCC in our cohort. BCCs were more common in the patients whose cSCCs were located in the trunk area or upper extremity. Subramaniam et al. reported that cSCCs occurred on body sites frequently exposed to sunlight, whereas BCCs occurred more often on sites of infrequent exposure, supporting the theory that lower doses of UV radiation can give rise to BCCs rather than cSCCs [24]. Furthermore, the patients in our cohort whose cSCC was located on an upper extremity were slightly more likely to have CM than those with their cSCC in other locations. In the light of these findings, cSCCs on the trunk or upper extremity could be considered signs of excessive UV exposure (with also high intermittent patterns) or an otherwise susceptible condition in the patient to be indicative of an increased risk of a variety of cutaneous malignancies.

Limitation of the study was that the information on the tumours was collected retrospectively, which meant that the numbers of premalignant lesions and their anatomical distribution could not be recorded nor could the data on the timeline on which these or other neoplasms emerged. Likewise, as the cohort was drawn from a single hospital, the results cannot be generalized.

In conclusion, a large subset of cSCC patients have a lifelong risk of developing multiple skin cancers and are in need of long-term follow-up and repeated courses of treatment. Counselling on sun avoidance and sun protection measures are essential to limit the increase in the number of types of skin cancer in the future. It is extremely important to examine the patient’s whole body at follow up visits [25]. The treatment and follow-up of patients with photo-damaged skin, a sign of increased skin cancer risk, must be recognised as a future health care challenge.
